# Ambient assisted living for frail people through human activity recognition: state-of-the-art, challenges and future directions

**DOI:** 10.3389/fnins.2023.1256682

**Published:** 2023-10-02

**Authors:** Bruna Maria Vittoria Guerra, Emanuele Torti, Elisa Marenzi, Micaela Schmid, Stefano Ramat, Francesco Leporati, Giovanni Danese

**Affiliations:** ^1^Bioengineering Laboratory, Department of Electrical, Computer and Biomedical Engineering, University of Pavia, Pavia, Italy; ^2^Custom Computing and Programmable Systems Laboratory, Department of Electrical, Computer and Biomedical Engineering, University of Pavia, Pavia, Italy

**Keywords:** human activity recognition, ambient assisted living, wearable systems, frail people, deep learning

## Abstract

Ambient Assisted Living is a concept that focuses on using technology to support and enhance the quality of life and well-being of frail or elderly individuals in both indoor and outdoor environments. It aims at empowering individuals to maintain their independence and autonomy while ensuring their safety and providing assistance when needed. Human Activity Recognition is widely regarded as the most popular methodology within the field of Ambient Assisted Living. Human Activity Recognition involves automatically detecting and classifying the activities performed by individuals using sensor-based systems. Researchers have employed various methodologies, utilizing wearable and/or non-wearable sensors, and employing algorithms ranging from simple threshold-based techniques to more advanced deep learning approaches. In this review, literature from the past decade is critically examined, specifically exploring the technological aspects of Human Activity Recognition in Ambient Assisted Living. An exhaustive analysis of the methodologies adopted, highlighting their strengths and weaknesses is provided. Finally, challenges encountered in the field of Human Activity Recognition for Ambient Assisted Living are thoroughly discussed. These challenges encompass issues related to data collection, model training, real-time performance, generalizability, and user acceptance. Miniaturization, unobtrusiveness, energy harvesting and communication efficiency will be the crucial factors for new wearable solutions.

## 1. Introduction

Ambient Assisted Living (AAL) refers to the use of Information and Communication Technologies (ICT), assistive devices, and sensor network technologies to support, monitor and enhance the quality of life for individuals, particularly older adults, or people with disabilities, within their daily living and working environment. The primary goal of AAL is to provide individuals with increased independence, autonomy, and safety by incorporating technological solutions into their surroundings. These solutions can assist individuals in various activities of daily living, such as managing their health, monitoring their safety, and improving their social interactions (Blackman et al., 2016; Stodczyk, 2020). One significant aspect of AAL is subject monitoring, which involves the continuous and unobtrusive tracking of an individual’s activities, health parameters, and environment to ensure their safety and provide timely assistance when needed. Subject monitoring utilizes various sensors to collect data and analyze patterns, enabling caregivers and healthcare professionals to gain valuable insights into an individual’s daily routines, health conditions, and potential risks. The choice of sensors can be made among two main groups: wearable and non-wearable sensors. The first one could be incorporated on clothing or worn by the user like accessories. Non-wearable sensors, on the other hand, are strategically placed on furniture, appliances, walls, doors, and other objects throughout the home. By integrating both types of sensors, through a so-called hybrid approach, a comprehensive monitoring solution can be created to effectively and efficiently monitor the subject (Calvaresi et al., 2017; Clapés et al., 2018).

**Figure 1:**
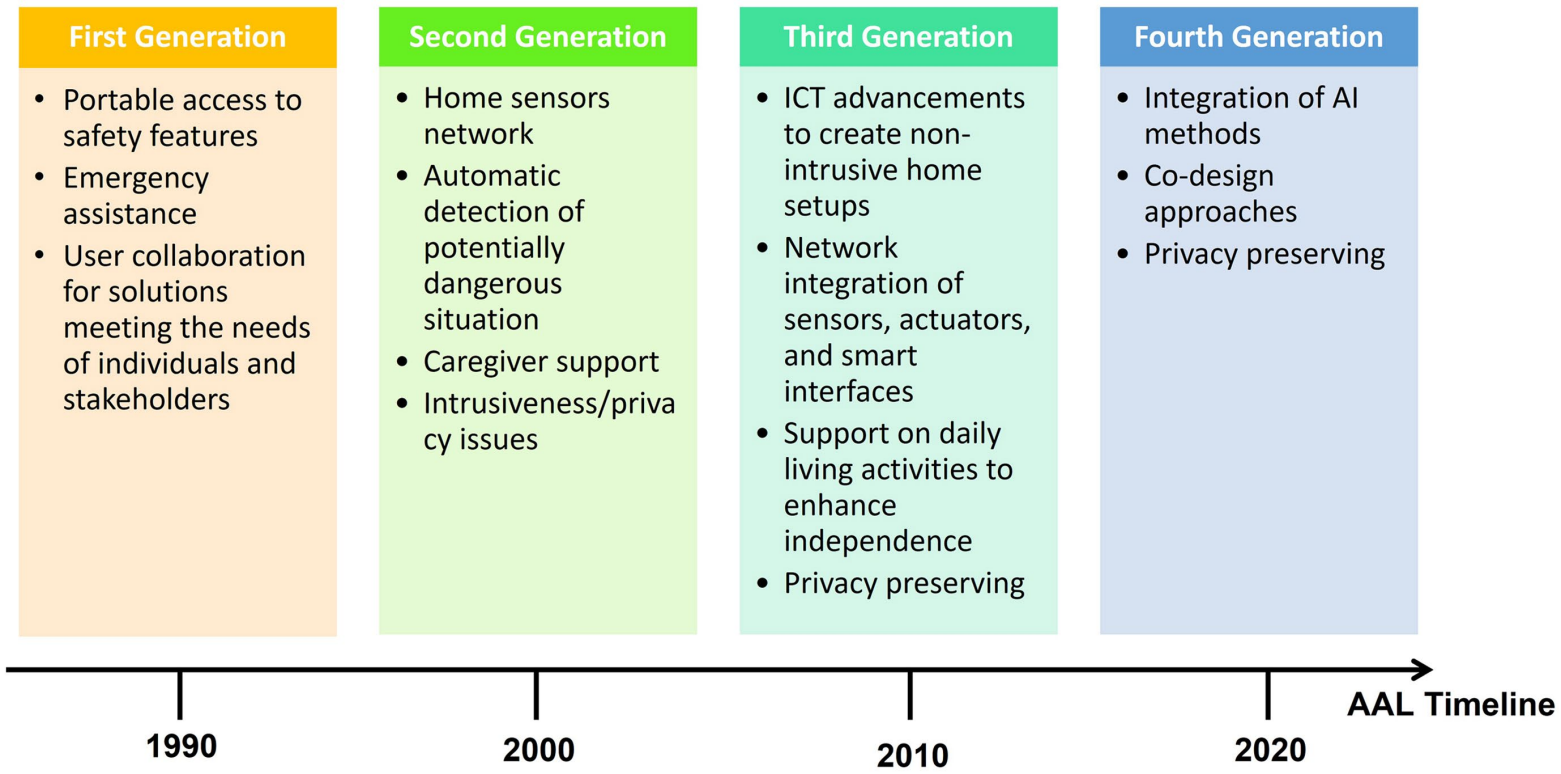
Evolution of AAL systems: four generations from the 1990s to the present day.

The work of Aleksic et al. proposed a subdivision of AAL systems for subject monitoring into four distinct generations (see Figure 1) based on technological variations, highlighting the application of ICT, stand-alone assistive devices, and technologies for indoor environments within individuals’ daily living and working environments (Al-Shaqi et al., 2016). These AAL systems actively encourage healthy lifestyles, contribute to disease prevention through personalized risk assessment and continuous monitoring, and primarily cater to frail individuals, by offering continuous support and actively promoting their independent and healthy living (Blackman et al., 2016; Calvaresi et al., 2017; Stodczyk, 2020; Cicirelli et al., 2021):

- *First Generation of AAL Systems*: the first generation of AAL systems primarily consists of alert and alarm systems using pendant or button devices worn by the monitored individuals. In the event of a dangerous situation, the individual would activate the button or pendant to send an alarm signal to a call center or caregiver. Examples of such solutions include the Salvalavita Beghelli^1^ and LifeAlert.^2^ While these devices offer several benefits, they also have specific limitations. For instance, individuals may be physically or mentally incapacitated, making them unable to trigger the alarm. Additionally, there are issues with individuals forgetting to wear or recharge the device.
- *Second Generation of AAL Systems*: the second generation of AAL systems involves more technologically advanced devices, installed in indoor spaces, incorporating sensors capable of automatically detecting dangerous conditions and triggering appropriate responses without relying on user activation. However, a weakness associated with this generation is that some users may perceive it as intrusive.
- *Third Generation of AAL Systems*: the third generation of AAL systems expands through advancements in ICT, introducing a more comprehensive concept of AAL. These systems encompass sensors designed to detect potentially dangerous situations and proactively prevent adverse scenarios, actuators providing support to the assisted individuals, and smart interfaces delivering information, assistance, and encouragement. The aim is to create minimally intrusive home setups comprising multiple sensors, actuators, and computing systems. These systems not only monitor the home environment but also track vital signs, changes in habits and activity patterns of frail individuals, and facilitate the execution of daily living activities (Mainetti et al., 2016).
- *Fourth Generation of AAL Systems*: the fourth generation of AAL systems incorporates Artificial Intelligence (AI) algorithms for data analysis within AAL solutions. These intelligent systems have the ability to learn from data and evolve over time, offering personalized assistance and support. The co-design approach is embraced, involving end-users, caregivers and stakeholders to create users-centered and inclusive solutions (Siriwardhana et al., 2019; Bansal et al., 2021; Sophia et al., 2021; Gulati and Kaur, 2022; Rupasinghe and Maduranga, 2022; Warunsin and Phairoh, 2022).

### 1.1. Related works

By combining AAL with subject monitoring, it becomes possible to create personalized and proactive care solutions, promoting independent living while offering a safety net for vulnerable individuals. Subject monitoring can be conducted in both outdoor and indoor environments. Outdoor environments expose frail people to various risks, such as falls, extreme temperatures, and potential wandering or confusion in individuals with early symptoms of dementia. In this context, AAL systems aim to provide support to frail individuals by facilitating route checking, anomalous behavior recognition, motion activity evaluation, and other relevant functionalities (Fernandes et al., 2020; Lee, 2021). Since wearables are the only devices that can be employed outdoors, they acquire a fundamental importance. Smartwatches and smart wristbands are the most commonly used devices, while Inertial Measurement Units (IMUs) are typically the sensors chosen (the same used in indoor scenarios) (Bhargava et al., 2017; Iadarola et al., 2022). In literature, other solutions have been proposed that address alternative or complementary approaches (Sokullu et al., 2019; Kenfack Ngankam et al., 2020; Rupasinghe and Maduranga, 2022): for example, Global Navigation Satellite System (GNSS) sensors for outdoor localization (Junior et al., 2023); instrumented insoles (commercial or customized) as an aid for gait detection and consequently for pointing out unsteady walking or falls (Cristiani et al., 2014; Sivakumar et al., 2018) and sensors mounted on the body of a walker as a low-cost solution for people with limited mobility (Ding et al., 2022). Conversely, indoor scenarios present frail individuals with risks closely associated with their living spaces.

Notably, the indoor environment has been identified as a significant contributing element to falls (Lee, 2021), which are attributed to factors such as uneven or slippery floor surfaces (including carpets and mats), tripping obstacles, inadequate lighting, poorly designed or maintained stairs without handrails and unsuitable furniture. These criticalities increase the likelihood of tripping, falling, or slipping for frail individuals. Additional hazards arise from the absence of safety or preventive devices, such as night lights and grab rails (Carter et al., 1997; Lee, 2021). Indeed, the requirements of monitored subjects can vary significantly across different indoor scenarios. In private homes, where individuals live alone or with a caregiver, the primary focus of monitoring is on preventing domestic accidents and delivering essential healthcare services. On the other hand, in retirement residences where multiple individuals share common spaces, subject monitoring systems are primarily designed to facilitate group activities and controlled physical exercises (Nastac et al., 2019; Cicirelli et al., 2021).

Adapting the monitoring approach to suit the specific needs and dynamics of each indoor setting is crucial. This ensures that monitored subjects receive personalized support and care tailored to their circumstances. Additionally, it is important to recognize that AAL systems cannot remain static, as people’s needs and habits evolve over time, along with the parameters that need to be observed. Consequently, data analysis methodologies must account for the evolving nature of these systems, allowing for the possibility of dynamically weighting or customizing certain parameters over others (Cicirelli et al., 2021). By embracing flexibility and adaptability, AAL systems can continue to provide effective and relevant support to individuals in various indoor environments.

The selection of appropriate sensors considers multiple factors, including the specific objectives of the AAL system, sensor cost, intrusiveness, user acceptability, and privacy concerns. However, more complex sensor networks, comprising environmental sensors, object sensors, cameras, and wearable sensors constitute the foundation of indoor AAL. The living facilities may be equipped with an array of interconnected sensors and actuators, enabling remote control and capable of detecting various environmental parameters such as door openings and room brightness. These sensors are strategically deployed to monitor the daily activities of individuals, ensuring security and safety. The selection of appropriate sensors considers multiple factors, including the specific objectives of the AAL system, sensor cost, intrusiveness, user acceptability, and privacy concerns. Communication protocols, such as ZigBee, Bluetooth, ZWave, USB, Ethernet, among others, are utilized to interconnect sensors, actuators, and smart devices throughout the environment (Tazari et al., 2011; Lloret et al., 2015; Babangida et al., 2022). Typically, raw or pre-processed data from sensors are transmitted to a collection center, either local or remote, where they undergo integration and analysis using robust algorithms (Plentz and De Pieri, 2018). A thorough and reliable data analysis becomes crucial in indoor scenarios equipped with automatic dangerous situation detection or capable of requesting help triggering alarms to third parties.

In the field of AAL, Human Activity Recognition (HAR) has emerged as a valuable tool with multifaceted utility. Within the AAL domain, HAR presents a range of solutions aimed at enhancing the quality of life of frail individuals (elderly and/or disabled people) and maintaining improved health and independence. Additionally, it provides also support to caregivers and medical professionals. HAR has garnered substantial interest as a prominent field of study in recent times. HAR methodologies are devised with the objective of autonomously detecting and classifying individuals’ routine activities within defined contexts. Depending on the task and the employed technologies, two main methodologies are commonly adopted. The first approach relies on a threshold analysis method, which can suffice for triggering alerts when detecting dangerous events (Zdravevski et al., 2017; Al Machot et al., 2018a,b; Cicirelli et al., 2021). The second and more recent approach (see Figure 1, fourth generation), employs Artificial Intelligence (AI) solutions such as Machine Learning (ML) and Deep Learning (DL) algorithms for HAR (Aggarwal and Ryoo, 2011; Wang J. et al., 2019).

Among the various possible applications, human activities can be classified into four distinct groups based on the involvement of various body parts (Jegham et al., 2020; Minh Dang et al., 2020):

- *gestures* involve basic actions carried out by different parts of the human body, including hand gestures like the “okay” gesture or “thumbs up” gesture;
- *actions* refer to a collection of fundamental movements executed by an individual, such as walking, standing, sitting, running, and other similar activities;
- *interactions* encompass not only activities involving interactions between two individuals, but they can also involve the relationship between a person and an object. Examples of interactions include playing a guitar or hugging another person;
- *group of activities* are the most intricate category as they primarily involve a combination of gestures, actions, and interactions. Examples of group activities include group meetings or group walking, where multiple individuals engage in coordinated actions and interactions.

Detailed framework of the HAR process will be discussed in the upcoming section. Finally, the aim of this work is to provide an overview of recent literature of AAL systems focusing on HAR from a technological perspective, tackling emerging evidences, challenges and future directions (Stavropoulos et al., 2020).

Papers have been revised by searching published works in the last 10 years in the following databases: IEEE Xplore, PubMed, Scopus. A series of keywords have been used, alone or in combination: ambient assisted living, ambient assisted living technology, healthy ageing, human activity recognition, ambient sensors, wearable sensors, wearable technology, activity monitoring, machine learning, deep learning, frail person.

The rest of the paper is organized as follows: Section 2 describes the Monitoring Solutions, focusing on the main components of an AAL system and detailing wearable and non-wearable sensors. Section 3 presents the processing chain, outlining the most relevant methodologies for data acquisition, processing and analysis. Section 4 proposes a critical discussion that also addresses future directions of this research field.

## 2. Monitoring solutions

Monitoring solutions in AAL systems can be developed using a variety of technologies, depending on the specific application domains and requirements. Specifically, in the field of HAR different solutions can be employed (Cicirelli et al., 2021; Ranieri et al., 2021). The upcoming session will provide a detailed description of wearable and non-wearable sensor solutions for HAR within the context of AAL in indoor environments.

### 2.1. Wearable sensors

Wearable devices typically consist of small sensors that can be integrated into cloths, rings, shirts, watches or other garments and devices. Such sensors gather body and context information to be locally processed or directly transmitted, mainly wirelessly through appropriate communication protocols, to a central processing unit of an AAL system (Kumari et al., 2017).

In the last few years, most wearable devices have been miniaturized and have optimized their power consumption (Cicirelli et al., 2021). Wearable devices, especially fitness trackers, wristbands and smartwatches (Andò et al., 2016; Lutze, 2018; Xie et al., 2020), have various built-in/integrated sensors such as accelerometers, gyroscopes and orientation sensors. Smartphones represent an additional solution for their characteristics of cost effectiveness and high number of embedded sensors (Ramanujam et al., 2021). Moreover, smartphones’ embedded sensors can be used alone or in combination with other wearable technology to evaluate posture and activities and to prevent falls, together with biological and behavioral monitoring (Haghi et al., 2019; Badgujar and Pillai, 2020; Nouredanesh et al., 2020). In this review, since users take their smartphone with them almost everywhere, even though they are not always in direct contact with the body, they are considered wearable devices at the same level as wristwatches, rings, glasses and necklaces, as opposed to environmental sensors and cameras. Apps for recording the device’s sensors data can be run on all commercial operating systems (Android or iOS) and they can be combined with commercial smartwatches, self-developed smart bands or devices like Shimmer nodes (Sanchez-Comas et al., 2020). However, not all applications provide an integration with smartphones; instead, many studies considered custom-developed solutions of electronic components. A significant number of works developed technologies in the laboratory, whereas fewer studies used commercial devices. Inertial sensors are the most common wearable elements used for HAR in AAL; in some cases, accelerometers (Tay et al., 2023), gyroscopes, magnetometers, temperature and object sensors may be applied, mainly worn on the waist or the hip (Pierleoni et al., 2019; Sarabia-Jácome et al., 2020).

Identification of the user’s position can be obtained even with passive RFID sensors or Bluetooth Low Energy (BLE) technology, that paved the way to the Bluetooth Smart for wearable devices (Ciabattoni et al., 2019; Paolini et al., 2019; Bilbao-Jayo et al., 2021). Also, wearables usually commercialized for fitness purposes have been evaluated for elderly wellbeing in the AAL domain (Piwek et al., 2016; Seneviratne et al., 2017; Cedillo et al., 2018; Surendran et al., 2018; Stavropoulos et al., 2020).

The majority of HAR systems in AAL are dedicated to the identification and management of falls, as confirmed by literature (Dhiman and Vishwakarma, 2019). In such systems, wearables are one of the key elements due to their mobility, portability, cost and availability and several studies have been conducted, mostly using a single wearable device (Wang X. et al., 2020). Even in this context, inertial sensors represent a large percentage of the research, whereas only a minority deployed other solutions (Bourke et al., 2007). Although Shimmer nodes, smartphones and smart watches often contain sensors like magnetometers, such elements were not normally used to detect falls; indeed, the only sensors embedded in smartphones used for this purpose are accelerometers and gyroscopes (Shi et al., 2016; Islam et al., 2017; Medrano et al., 2017; Chen et al., 2018).

Even combining multiple sensors into a single framework can provide valuable data for meaningful and complex predictions, thus achieving a more versatile, robust and trustworthy wearable system for HAR purposes. Moreover, commercial tools are widely used, such as Samsung Galaxy Gear Live (Faye et al., 2017), Microsoft Band 2 (Garcia-Ceja et al., 2018) and Intel Basis Peak (Kang and Larkin, 2017), as well as other alternatives like Empatica E3 (Clapés et al., 2018), Fitbit (Kang and Larkin, 2017), and Google Glass (Shewell et al., 2017; Clapés et al., 2018).

It is worth noticing that most of the time, wearable technology alone would be sufficient to assess activity recognition in indoor environments and AAL systems. This is an important advantage, combined with their low cost, portability and unobtrusiveness. However, a hybrid approach combining wearable and non-wearable sensors often overcomes possible drawbacks due to users not wearing the device correctly, thus leading to low quality signals, not being comfortable in putting on a wearable that could excessively stand out, forgetting to charge it or even to wear it continuously.

### 2.2. Non-wearable sensors

Non-wearable sensor solutions for HAR encompass devices or systems that can detect and analyze human activities without needing to be directly attached to the body. These solutions play a crucial role in the functionality and effectiveness of AAL systems. Operating in a passive manner, these sensors autonomously monitor room occupants without the need for manual intervention. This eliminates the need for users to carry additional devices during their daily activities.

One example of a non-wearable sensor solution is radio-frequency-based systems, as demonstrated by (Fan et al., 2020). These systems utilize the analysis of radiofrequency (RF) signal reflections to monitor various activities performed by individuals. In this study, Radio Frequency sensors were embedded directly in the floor to capture the everyday activities of residents (Fan et al., 2020). As part of radiofrequency-based systems, radar and ultra-broadband technologies, as well as automotive-derived solutions, also emerged as interesting approaches for human activity recognition (Guendel et al., 2021; Senigagliesi et al., 2022).

Furthermore, contemporary monitoring and behavior analysis tasks can be facilitated by diverse image-based technologies.

Nowadays, low-cost cameras have emerged as viable options for monitoring individuals’ daily activities, ensuring their well-being (Geman and Costin, 2014; Cebanov et al., 2019; Minh Dang et al., 2020; Ryselis et al., 2020; Bouchabou et al., 2021; Chen et al., 2021; Raeis et al., 2021). These devices enable continuous monitoring of individuals without requiring their active involvement. These include RGB, Depth, and RGB-D cameras, as well as IR (infrared) array sensors, known as thermal cameras.

RGB cameras, being widely available and affordable, provide information about the shape, color, and texture of the scene (Zerrouki et al., 2018). However, they have some drawbacks such as a limited field of view, complex calibration procedures, sensitivity to environmental variations (e.g., lighting conditions, type of illumination and cluttered background) and privacy concerns. To address this latter issue, depth cameras offer distance information from the sensor to elements in the scene, capturing detailed spatial information while maintaining heightened privacy protection (Jayaraj et al., 2019; Beddiar et al., 2020). Depth sensors also exhibit superior resilience to variations in illumination, color, and texture compared to RGB devices. However, noisy measurements can occasionally affect accurate object or subject identification, necessitating data processing and refinement. In recent years, low-cost devices integrating RGB and depth sensors, such as Kinect and Intel RealSense systems, have been employed as environmental sensors in AAL systems. Another alternative, IR array cameras, measure thermal energy emitted by the human body or other objects (Mashiyama et al., 2015; Spasova et al., 2016; Karayaneva et al., 2023). These low-resolution IR arrays offer advantages such as privacy preservation, low power consumption, insensitivity to ambient lighting variations, operation in complete darkness, fast response time, easy deployment, and straightforward image processing.

All these devices suffer from subject occlusion, which occurs when certain body parts of the subject are hidden or obscured by other objects or body parts within the room, leading to incomplete or inaccurate tracking of the subject’s movements. To overcome the occlusion limitation, a practical solution is to employ a multiple camera setup that covers various areas of the room from different viewpoints. By using multiple cameras, the chances of occlusion can be reduced, as different cameras capture different perspectives of the scene. This approach allows for a more comprehensive view of the subject’s activities and improves the accuracy of tracking. However, it is important to note that using multiple cameras increases HAR systems’ costs and requires synchronization among them to ensure proper coordination and alignment of the captured data. Synchronizing the cameras enables the seamless integration of the captured images or depth data, allowing for a more complete understanding of the subject’s movements and activities.

In the context of monitoring human activities, sensors can be also embedded in everyday objects within the environment. Contrary to wearable sensors, which directly measure human activities, these sensors enable the detection of movements and activities through the usage of specific objects, providing valuable insights into the daily lives of individuals (Bassoli et al., 2017; Rafferty et al., 2017). For instance, sensors can be integrated into furniture items such as carpets, beds, fridges, and more, allowing for unobtrusive monitoring of daily living activities. Presence statistics of users in different spaces can be gathered by monitoring the sensors embedded in furniture. Power meters can be employed to track appliance usage (Bianchi et al., 2019), such as monitoring TV sets, while smart pill box devices assist in checking medication intake (Keum et al., 2019). Roland et al. proposed the installation of an accelerometer attached to a smart drinking cup to efficiently identify the user’s drinking movement (Roland et al., 2018). Bassoli et al. installed sensors directly on the furniture of the subject’s house for HAR. Pressure pads are used to monitor bed or chair occupancy, while sensors inside the fridge provide indirect information about feeding habits (Marenzi et al., 2012, 2013a,b; Bassoli et al., 2017). Chaccour et al. developed a smart carpet with piezoresistive pressure sensors to detect falls of the inhabitant (Chaccour et al., 2015; Singh et al., 2020).

Everyday object sensors offer a less invasive and privacy-friendly alternative to cameras, as they are designed to specifically recognize human activities related to the intended use of the object. By focusing on that, these sensors can provide valuable insights and functionality while minimizing potential privacy concerns. Everyday object sensors can detect interactions with household appliances or devices, such as opening a refrigerator, without capturing or storing detailed visual information of individuals. Yue et al. proposed an RF-based system that accurately monitors sleep postures overnight in the user’s own house. By analyzing RF reflections and distinguishing them from other signals, the system can identify different sleeping postures such as supine, left side, right side, and prone (Yue et al., 2020). This approach can help alleviate privacy concerns while still enabling the development of innovative and convenient technologies that enhance user experiences in a responsible and respectful manner.

## 3. HAR processing chain

**Figure 2:**
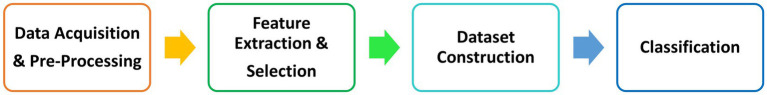
Processing chain illustrating the general steps of HAR.

Irrespective of the specific type of human activity being classified, the process of HAR typically adheres to a standard framework comprising several distinct phases. These phases are summarized in each block of Figure 2 and detailed in the following paragraphs, keeping the same names and order as in the Figure 2.

### 3.1. Data acquisition and pre-processing

Sensors and devices are characterized by outputs that are either punctual values (i.e., pixels in an image) or temporal series (i.e., position, acceleration). HAR applications could rely on raw data or need further pre-processing steps to enhance the signal quality (i.e., noise reduction, data normalization, segmentation) and/or to obtain derived data. For instance, from data captured by RGB-D cameras it is possible to estimate the position over time of specific points on the human body, often corresponding to anatomical points of repère or joints with respect to a specific coordinate system typically intrinsic to the device. This data processing stage is called skeleton tracking. Moreover, joint angles can be obtained starting from body joint positions (Jegham et al., 2020). Notably, in the context of AAL, it is common practice to store and analyze the skeleton tracking data instead of RGB or depth data to prioritize privacy preservation (Gasparrini et al., 2014; Tu et al., 2018; Li and Sun, 2021; Srimath et al., 2021). The pre-processing stage aims at noise reduction, data normalization and segmentation.

#### 3.1.1. Noise reduction

Raw, noisy signals adversely affect signal segmentation and possible further computing steps (Deep et al., 2020). Noise can be removed in several ways: linear, averaging low-pass and high-pass filters (Amin et al., 2016). In some specific situations (e.g., inertial data and skeletal data), Kalman filters, moving average or adaptive filters are useful to correct distorted signals (Jeong et al., 2017; Beddiar et al., 2020; Ahad, 2021). The choice of an appropriate filter is critical, since it affects the quality of the filtered signals and of the successive steps of the processing chain. The Signal-to-Noise Ratio (SNR) is a crucial parameter in the identification of the most appropriate filter, together with the correlation coefficient (R) between the filtered and the reference signals, the cut-off frequency, the waveform delay, the filter size and the window length. The choice of the most suitable filter has to be a compromise among all these parameters. The components of the target signal that fall within the stop band of the filter are lost. At the same time filtering a signal introduces a delay (waveform delay), i.e., the output signal is shifted in time with respect to the input. This factor plays a critical role, especially when the responsiveness of the application is mandatory. The filtering of the signal at the same time as its acquisition is important in order not to accumulate delay between the filtered and raw signal. In other words, if the complete processing chain is designed to identify a potentially dangerous situation, the time shift should be compatible with this task, to ensure a prompt detection. Finally, a filter operates by allowing a specific range of frequencies to pass through. For instance, since frequency range of human activity is usually about 0–20 Hz (Antonsson and Mann, 1985; Grossman et al., 1988), the cut-off frequency is usually equal to at least twice such value (Castro et al., 2016; Wang et al., 2017; Ma et al., 2018; Minh Dang et al., 2020).

#### 3.1.2. Data normalization

Data normalization is characterized by scaling or transforming the acquired data. This step is often necessary in HAR scenarios where data originated from different types of sensors and/or from people with various anthropometric characteristics. According to the type of data (e.g., RGB or depth, temporal or skeleton data) several normalization methods can be implemented (Pires et al., 2020; Ahad, 2021). Common normalization methods are min-max, mean, standardization and scaling to unit length. The first method scales the data in their maximum and minimum range: the minimum value is subtracted from each data point and the result is divided by the data range. In the second one the mean of all data samples is subtracted from the data vector, and the result is divided by the difference between the maximum and minimum samples. In the standardization method, the mean value of all data samples is subtracted from the data vector, and the result is divided by the standard deviation value. Finally, the last normalization technique scales all the data with respect to the sum of all elements of the data vector (Patro and Sahu, 2015; Mistry and Inden, 2018; Narkhede, 2019; Ahad, 2021).

When considering skeletal tracking data instead, there are also two other types of normalization methods. The first one is the Bounding-box normalization (referring to the border’s coordinates that enclose the subject), in which all skeleton 3D joints coordinates are normalized using the maximum side-length of the bounding box of the skeleton (Cippitelli et al., 2016; Liu et al., 2020). In the second method data are normalized by dividing the 3D coordinates of the skeleton with respect to the length of a specific body segment (i.e., head, neck, torso and so on) or by the subject height. For example (Cippitelli et al., 2016), scaled joint position dividing each value by the Euclidean distance between the neck and torso joints.

#### 3.1.3. Data segmentation

Data segmentation is strongly related to the type of data. When dealing with temporal sequences, it consists of partitioning the data into time windows. Otherwise, when RGB or depth images are analyzed, the segmentation involves the separation of the selected target subject in the scene from the background. The data subdivision in time windows is principally done to overcome the limitations due to the difference between the duration of the action and the sampling rate imposed by the data acquisition device (Minh Dang et al., 2020; Ahad, 2021). The data segmentation can be categorized into Fixed size Non-overlapping Sliding Window (FNSW), Fixed-size Overlapping Sliding Window (FOSW) or Event-Driven Sliding Window (EDSW) (Bulling et al., 2014; Ahad, 2021). In FNSW and FOSW segmentations, the number of samples included in each window is fixed. The difference is that in FOSW consecutive analysis blocks of the time data are overlapped by the designated percentage of the time record, while in FNSW there is no overlapping. Concerning EDSW, it differs from the others since the number of samples included in the time window is not fixed (i.e., it features a variable window size) (Devanne et al., 2019). Generally, the window size has to always be carefully established to comprehend an adequate number of samples, at the same time avoiding prolonged execution times. When the HAR process is part of a system aiming at monitoring a person in the AAL environment, in which the promptness of the recognition is mandatory, smaller window segmentation is suitable (Buzzelli et al., 2020). It also reduces the complexity and the computational time of the HAR process. The overlapping technique can handle the transition between human activities more accurately, i.e., the transitions between sitting and standing postures, or between walking and running (Torti et al., 2019; Buzzelli et al., 2020; Khan and Ghani, 2021; Guerra et al., 2022, 2023). In case of RGB image, or depth image, the segmentation process is implemented using two different approaches: namely, the background subtraction and the foreground extraction. The first one consists in the extraction of the body silhouette in an image sequence captured from a static camera by comparing each incoming frame with a background model. A crucial step of this technique is to obtain a stable and accurate background model. The second one is recommended when the images are acquired by a moving camera, and it consists in the computation of the difference between consecutive image frames. The foreground extraction is more challenging than the background subtraction because, in addition to the motion of the target object, it also needs to consider the motion of the camera and the change of background (Ke et al., 2013; Babaee et al., 2018; Minh Dang et al., 2020; Mliki et al., 2020).

### 3.2. Feature extraction and selection

The feature extraction procedure consists in the definition of a set of parameters able to discriminate the activities to be classified. Based on the given data nature and characteristics, the features can be divided into several categories: time-domain, frequency-domain and kinematic features (Dhiman and Vishwakarma, 2019; Sousa Lima et al., 2019; Ahad, 2020, 2021). The time-domain features are usually defined to describe the data amplitude variation and distribution over time (for instance mean, variance and kurtosis). On the other hand, the frequency-domain features show the distribution of signal energy (i.e., Fast Fourier Transform, entropy and power spectral density). Kinematic features include all the characteristics of the subject’s movements, acceleration and posture (joints positions and angles). The kinematic features describe geometric relations between body joints (Müller et al., 2005; Guerra et al., 2020).

In the case of images, usually global and local features are computed. The first ones describe the image frames as a whole, providing different types of information (spatial, temporal, frequency) (Ke et al., 2013). Local features extract information around a set of interest points or describe a selected image region, through techniques like histograms of oriented gradients (Aly and Sayed, 2019).

After feature extraction, the relevant ones are selected to achieve dimensionality reduction by finding the smallest subset of features which efficiently defines the data for the given problem (Blum and Langley, 1997; Chandrashekar and Sahin, 2014; Jindal and Kumar, 2017; Ayesha et al., 2020). It can be accomplished using different methods, such as filter, wrapper, embedded, and the more recent hybrid approach (Blum and Langley, 1997; Minh Dang et al., 2020; Zebari et al., 2020). Filter methods measure the relevance of features using statistical standards for evaluating a subset, they process data before the classification occurs and are independent from the latter. The features are ordered according to the ranking of importance (computed with suitable score metrics) and those below a certain threshold are removed. Among the different algorithms, the most used are: ReliefF, statistical techniques such as Principal Component Analysis, Independent Component Analysis, Neighborhood Component Analysis and Correlation Based filter (Suto et al., 2016; Alzahrani et al., 2019; Siddiqi and Alsirhani, 2022). Wrapper method selects the optimal features subset evaluating alternative sets by running the classification algorithm on the training data. It employs the classifier estimated accuracy as its metric (Bhavan and Aggarwal, 2018; Zebari et al., 2020). The most used iterative algorithms are the Recursive Feature Elimination with Support Vector Machine, the Sequential Feature Selection algorithm and the Genetic Algorithm (Liu and Shao, 2013; Guerra et al., 2022). Compared to the filter method, the wrapper method achieves better performance and higher accuracy, nevertheless it increases computing complexity due to the need to recall the learning process for each feature set considered (Jindal and Kumar, 2017; Zebari et al., 2020). In the embedded method, as the name suggests, the selection occurs within the learning algorithm. The most common are the tree-based algorithms like, for example, the Random Forest and the Decision Tree. Embedded methods can be used in multiclass and regression problems and compared to a wrapper method, it is computationally more effective while retaining similar performance (Negin et al., 2013). Finally, the hybrid approach combines filter and wrapper methods to achieve the benefits of both. Usually, the filter technique is first applied to reduce the search space and then, a wrapper model is used to acquire the best subset (Peng et al., 2010).

### 3.3. Dataset construction

Dataset construction concerns the process that divides the acquired data into training, validation and test sets. Generally, a set of data is required to train the classification model and a set of validation data is used to evaluate the performance of the model during training epochs for fine tuning the hyperparameters and to estimate if the model does not overfit, i.e., when a statistical model fits exactly against its training data at the expenses of its generalization abilities. Finally, test data, different from those involved in the training set, are used to evaluate the performance of the model (Bouchabou et al., 2021). The data contained in the training, validation and test could be described by labels also called classes. As will be stressed in the next Section, this operation is of crucial importance for the classification algorithms. In HAR tasks, the classes represent the type of activity to be recognized (i.e., walking, sitting, lying down, and so on) (Sathya and Abraham, 2013; Sindhu Meena and Suriya, 2020).

In HAR, three methods have been used to divide the data into training, validation and test set. In the first one, called cross-subject, the subjects are divided into two groups. The data of the first group are used for the training phase, whereas those of the second one are involved in the validation and test phase (Khan and Ghani, 2021). The cross-subject method aims at guiding the learning process of the AI model so that it becomes as robust as possible, in order to adapt it to the heterogeneity of the subjects. The second splitting method is characterized by randomly dividing the whole dataset based on a percentage such as 70-30%, 80-20%, and so on. The larger portion is fed for training the model where the other portion is kept for validation and test (Ahad, 2021). This is the most used splitting criteria in the general problems of classification algorithms and have been reported in HAR with success. Alternatively, when data are acquired by multiple cameras with different points of view, a cross-view method can be used. In this case the data coming from one or more cameras are used for the training phase and those of the remaining ones for the validation and test phase (Wang L. et al., 2020).

### 3.4. Classification

The most critical step of HAR systems relies on classification algorithms. In the literature, two main categories can be identified, namely threshold-based and AI methods. Furthermore, AI algorithms can be divided into ML and DL techniques. In the following, these methods are analyzed, highlighting the main advantages and issues in their application for HAR data analysis.

#### 3.4.1. Threshold-based methods

Threshold-based methods are the first introduced in the literature and they typically do not need feature extraction and selection. They are based on comparing the acquired values with a pre-defined threshold (Fetzer et al., 2018). If a signal value exceeds the threshold, then the algorithm identifies the targeted situation. More advanced threshold-based algorithms adopt adaptive thresholds (Madhu et al., 2021) or apply it on statistical indicators extracted from the original signals (Cola et al., 2017). Moreover, data fusion is employed when considering multiple sensors. A particular strategy uses only one of them to make a final decision: this is called partial fusion. An example is a fall detection system that employs a tri-axial accelerometer and an RGB-D camera: only when the measured signal exceeds a threshold, the camera is activated to capture the ongoing event (Kwolek and Kepski, 2014). One of the most important advantages of threshold-based algorithms is the low computational complexity. This allows the deployment of these algorithms directly on a small computation unit which typically also manages the data acquisition and pre-processing. Indeed, this solution is widely adopted for wearable devices which do not rely on external centralized processing (Jung et al., 2015; Cola et al., 2017). Concerning non-wearables, the preferred strategy is to send all the acquired data to a central host, which applies the threshold-based algorithm (Andò et al., 2016). The major drawback of these methods is the threshold selection since it depends on the monitored subject. Indeed, inter-subject movements show high variability and even the same person can perform a certain movement in different ways (intra-subject variability) depending on a multitude of factors, such as injuries or illness (Jegham et al., 2020). This affects the classification performance.

#### 3.4.2. Machine learning methods

In the last decade, ML methods have been widely explored for HAR since they can automatically extract high level features and produce more affordable results than threshold-based approaches. In particular, the best results have been achieved by Support Vector Machines (SVMs), Artificial Neural Networks (ANNs), K-Nearest-Neighbours (KNN) and Complex Trees (Oniga and Suto, 2014; Hemmatpour et al., 2017; Su et al., 2017). SVMs and KNN rely on the concept of instances. First, they create sets of example data, in which each set is related to a specific class. Then the distances between the new data and the example data sets are computed. The aim is to find the example set with the minimum distance from the new data. Finally, the class of the minimum distance set is given to the new data. ANNs are based on the structure of biological neural networks. They are composed of elementary computational units, which perform a weighted sum of the inputs and apply a nonlinear function. These are organized into layers as Multi-Layer Perceptrons (MLP) and are used to map input data into output classes. Finally, Complex Trees build a decision-making diagram with a tree structure. The tree structure is based on the attribute values of the input data. The classification is obtained following the tree structure until a leaf is reached. Later, single algorithms have been combined in the so-called ensemble learning with different strategies such as boosting, stacking, bagging and majority voting to enhance the classification quality (Hasan et al., 2022).

These algorithms have been used both for data acquired by wearable and non-wearables devices. In both cases, the data are acquired and sent to a central unit for the classification step (Sheikh and Jilani, 2023). Therefore, the main drawback is related to data transmission since the communication rate should be high enough to guarantee continuous monitoring. Moreover, this represents a critical issue for wearable devices since wireless communication is the main power consuming process.

Concerning classification performance, it is affected by the quality of the dataset. AI methods need to be trained on significant examples, which should be balanced among the different classes conceived in the target application. It is worth noticing that an unbalanced training set negatively affects the model performance. A recent trend is to exploit data augmentation strategies to create synthetic data both to increase the training set size and to balance it (Um et al., 2017; Mathur et al., 2018; Steven Eyobu and Han, 2018). The main drawback is related to the choice of the augmentation techniques, since synthetic data can differ too much from the real ones.

#### 3.4.3. Deep learning methods

DL emerged in recent years as the most powerful AI tool to automatically extract high level features and perform affordable classifications. The development of DL models has been enabled by the computing power offered by the technological evolution of devices such as multi-core processors and graphic processing units. Among the different DL methods used in HAR Convolutional Neural Networks (CNNs), Long Short-Term Memory (LSTM) cells and Gated Recurrent Units (GRUs) (Torti et al., 2019; Ronald et al., 2021; Poulose et al., 2022; Guerra et al., 2023; Sonawani and Patil, 2023) emerged as suitable solutions. CNNs are mostly used to deal with visual data, since they roughly imitate human vision. The data is processed as a grid-like topology through the convolutional operator. They can be used both for images and for time series. On the other hand, LSTM and GRUs are mainly used for time series analysis. In fact, their main feature is to learn time dependencies.

These methods have been initially used for data coming from non-wearable devices, especially when vision-based sensors were employed. However, they are gaining popularity also for wearable devices (Torti et al., 2019; Goh et al., 2021; Luwe et al., 2022).

### 3.5. Computing power constraints

An important aspect related to HAR for frail people is the time needed by the AAL system to detect possible dangerous situations. This time is strictly related to the computational complexity of the adopted algorithms and to the computing power of the devices which perform the processing chain.

Threshold-based methods are the lowest computing power demanding techniques, since they are based on simple comparisons with a fixed value. Even in the case of threshold computed at runtime and/or applied to statistical indicators, the computational complexity can be easily managed by standard microcontrollers and does not have a critical impact on processing times.

AI methods are characterized by a higher computational complexity than threshold-based techniques. In particular, DL methods feature the highest computational complexity, not only for their training, which is run off-line and may require multiple CPU and/or GPU, but also for their implementation as a classifier once all the weights are determined. Therefore, their processing is typically performed by a centralized unit for both wearable and non-wearable devices. However, recently some researchers have designed DL algorithms on low power devices suitable for wearable applications.

Recent and very complex solutions exploited ensemble learning also with DL algorithms (Kumar and Suresh, 2023) enhancing the classification performance of single techniques.

The increasing popularity of AI solutions has led to the development of various software tools, libraries, and frameworks that facilitate the implementation of these algorithms on devices with limited resources. An example of such a tool is TensorFlow Lite for microcontroller^3^ (TFLM), an open-source library designed to enable the implementation of AI methods (i.e., ANNs, LSTM, CNN and so on) on a wide variety of MCUs and Digital Signal Processors (DSPs). TFLM allows the execution of pre-trained algorithms developed using TensorFlow for on-device inference. Another prominent solution is X-Cube-AI, a software tool developed by STMicroelectronics.^4^ It offers a comprehensive environment for generating and optimizing AI algorithms developed using popular ML and AI frameworks such as TensorFlow,^5^ Keras,^6^ or PyTorch.^7^ X-Cube-AI is tailored for deployment on the STM32 family of MCUs, empowering developers to leverage familiar frameworks and simplify the integration of AI capabilities into their applications. In addition to these tools, there are cloud-based platforms like Edge Impulse, which provides a flexible environment for the development of AI models. Edge Impulse^8^ supports various embedded platforms, including MCUs and smartphones, enabling developers to create AI models that cater to diverse hardware constraints. NanoEdge AI Studio^9^ is another valuable tool that supports both learning and inference directly inside the MCUs. Notably, it offers the advantage of automatically selecting the most suitable machine learning libraries based on the provided data (Shumba et al., 2022).

Inference needs to be performed under real-time constraint, especially when a potentially dangerous condition needs to be identified. This means that the classification should be strictly performed prior to a fixed temporal deadline, which is defined by the acquisition time window. In other words, the system acquires a window of data and its pre-processing and classification should be performed before the end of acquisition of the following time window. The computational complexity of the algorithms and the computing units included in the system play a critical role in the real-time compliance (Avram and Pop, 2023; de la Cal et al., 2023; Gonçalves et al., 2023; Saliba et al., 2023; Zeng et al., 2023). Delayed or sluggish processing can hinder the effectiveness of AAL systems in providing timely assistance, which is crucial for ensuring the safety and well-being of individuals. Efficient algorithms and optimized implementations are necessary to overcome these constraints and enable real-time processing on resource-limited platforms. The computational unit controls the sampling and acquisition of data: usually this is performed by Commercial-Off-The-Shelf (COTS) low power and low cost microcontrollers, supporting interfaces and data transfer protocols (SPI, I2C…). When high speed, flexibility and control over the elements of the architecture are required, Field Programmable Gate Arrays (FPGAs) and Application-Specific Integrated Circuits (ASICs) can be used, with increased costs and higher time for development and/or production. The computational unit is equipped with initial signal conditioning and processing algorithms together with specific classification methods that perform data analysis for local decision making, real-time response and forwarding processed data to successive layers. Characteristics that can influence all these choices include power consumption, computational and storage capacity, complexity and results accuracy of the algorithms, privacy concerns and latency requirements. Pre-trained models using computationally efficient algorithms may be used for anomaly detection and the results can produce warnings or propose a course of action. Alternatively, a fraction of an ANN can perform partial on-device data processing, to forward only intermediate data, thus ensuring also the privacy of the user (Zhang et al., 2023). Lastly, after the elaboration and analysis of data, results, inferences, or alarms are packaged and forwarded to the upper layers for further processing or management (Shumba et al., 2022).

## 4. Discussion

This review aims at providing an overview on the application of HAR process in the context of AAL systems, underlining their potential to support independent living for frail individuals.

Recent advances in AAL technologies and the reduced cost of sensors have encouraged the development of technological environments to enable frail people to live healthier and more independent lives and to support caregivers, medical personnel, thus limiting hospitalization, promoting personalized therapy and enhancing wellbeing. To provide such services, an AAL system has to be able to understand the daily activities of its residents. In general, the choice of technologies used for HAR purposes can include wearable sensors (IMUs, smartwatches, smartphones…), non-wearable sensors (environmental devices, objects, and cameras), or a combination of the two (hybrid approach). The sensor selection can be made especially depending on the individual’s needs. Wearable sensors have numerous advantages, including their small size, low power consumption, direct acquisition of information on the subject’s activity and full respect of the subject’s privacy. At the same time, they also have some drawbacks. For example, they need to be worn by the subjects and to operate for long time periods. The latter could represent a significant problem for the monitored subject and for the battery life of the devices. Also, to fully capture the 3D motion associated with a human action, a single sensor may not be adequate. It may be necessary to utilize multiple configurations, thus increasing the intrusiveness of the devices worn by the subject (Wang Y. et al., 2019; Beddiar et al., 2020; Qiu et al., 2022). On the other hand, environmental and camera sensors offer the key advantage of being unobtrusive, as they do not require the individual to wear them. However, they also face certain challenges. One major issue is their reliance on infrastructure, which can limit their effectiveness in identifying specific movements or activities. Additionally, the utilization of environmental sensors is less frequent compared to wearable sensors due to higher costs and setup difficulties. Furthermore, similar to the wearable approach, this solution may not always be feasible as it requires users to interact with tagged objects or to remain within the environment where the sensors are installed. In particular, cameras suffer from drawbacks like occlusion, multiple-view variations and privacy issues (Minh Dang et al., 2020). A possible solution to handle occlusion relies on a multiple camera setup, even though these devices need to be synchronized among each other. Cameras are often perceived as the most intrusive technologies in terms of the privacy of the monitored individuals. The solution to this drawback may be RGB-D cameras, like the Kinect V2, which, through a data elaboration, are able to extract the “skeleton” of the subject from the depth image, i.e., the subject is represented as a set of body segments and joints, bypassing the need for using the RGB image for HAR purposes. These tools increase the person’s acceptance towards the assistive technology, since they ensure privacy preservation (Gasparrini et al., 2014). Among non-wearable devices, object sensors are the least invasive and the most respectful of users’ privacy, as they focus on recognizing human activities related only to the intended use of the object. For example, a smart cup recognizes drinking actions, and sensors embedded in cushions or beds identify specific sleeping postures.

To overcome the wearable and non-wearable limits a possible solution could be represented by a hybrid approach. Sensor fusion provides a more robust approach since multiple sensors may complement each other with their specific signals, producing a reliable system (Wang X. et al., 2020). Moreover, the likelihood of having missing data is progressively balanced out by increasing the number of sensors in the system. However, critical issues in AAL services stem from the integration of multiple technological types, mainly environmental and wearable sensors (Calvaresi et al., 2017). For instance, hybrid systems are characterized by sensors with different sampling frequencies. Thus, synchronization and interpolation of acquired data is mandatory for better correlation of output devices. Furthermore, some challenges can still be present; for example, security and privacy requirements need to be taken into consideration.

Independently from the adopted monitored system, HAR requires affordable processing chains to classify the target human activities. Typically, HAR should be performed meeting the real-time constraint, especially when frail people are monitored. While threshold algorithms are efficient for real-time processing, they may struggle with handling complex activities or adapting to dynamic contexts. These limits are overcome by AI methods, at the price of an increased computational complexity, which negatively impacts on classification time. For this reason, the optimal solution requires a suitable trade-off between classification quality and processing time to ensure real-time compliance. Common factors that determine real-time compliancy of a method are the computational complexity and the processing power of the system. It must be stressed that the computational complexity alone is not sufficient to determine if a method is real-time compliant. Indeed, the response time strictly depends on the processing power of the device. It is not trivial that very different processing units perform the same computation with significantly different times. Therefore, the choice of a suitable processing element covers a critical role in the real-time compliancy of a system. Moreover, datasets derived from real situations are not always available or sufficiently precise; in some cases, only simulated conditions are present, but this greatly compromises the results (Casilari and Silva, 2022). At the same time, prompt detection of dangerous conditions cannot be provided in some contexts.

Table 1 summarizes the type of activity, sensors, input data, datasets, main approaches and potential applicability in a real-life scenario, considered in the most relevant works in the field of Human Activity Recognition, already cited in the previous sections. It categorizes the papers into three main classes on the basis of their adopted technologies: wearable, hybrid and non-wearable solutions. In each row, among the labelled information previously reported, the type of activity is related to the target application of each proposed HAR system. Indeed, most of the works aim at recognizing falls and/or Activities of Daily Living (ADL), i.e., lying down, walking, sitting and so on. The input data type is labelled “Dynamic” in case of time series or “Static” otherwise. Concerning the datasets, publicly available ones present the corresponding reference paper, whereas custom Datasets report the number of involved subjects and the amount of performed activities (if one or more parameters are not available, the acronym N.A. is indicated). The “Real life scenario” column is related to the system applicability outside the laboratory setting. It is labelled with “YES,” in case of data acquisitions performed in an environment which considered not only ideal conditions (i.e., the subject is always directly in front of the camera or the falls are simulated only by young people).

**Table 1:** Summary of the analyzed literature.

Authors and year	Activity	Type of sensor	Input data type	Dataset	Approach	Real life scenario
Wearable solutions						
Andò et al. (2016)	ADL and Fall Detection	Tri-axial nano-accelerometer and gyroscope embedded in a smartphone	Static	Custom (10 healthy subjects; 6 activities)	Threshold-based Data Fusion	NO
Xie et al. (2020)	ADL and Fall Detection	Smart watch	Static	Custom (5 subjects; 9 activities)	Threshold-based SVM	NO
Badgujar and Pillai (2020)	Fall Detection	Tri-axial accelerometer	Static	SisFall (Sucerquia et al., 2017)	SVM and Decision Tree	NO
Nouredanesh et al. (2020)	Compensatory Balance Reactions (CBRs) for Fall Detection	IMU	Static	IMUFD (Aziz et al., 2017) and Custom (9 healthy subjects; 9 activities)	Random Forest	NO
Pierleoni et al. (2019)	Postural stability	IMU: tri-axial accelerometer, tri-axial gyroscope and tri-axial magnetometer	Static	Custom (6 healthy subjects; 3 activities)	Filter-based Data Fusion	NO
Sarabia-Jácome et al. (2020)	Fall Detection	Tri-axial accelerometer	Dynamic	SisFall (Sucerquia et al., 2017)	LSTM and GRU	NO
Bourke et al. (2007)	ADL and Fall Detection	Tri-axial accelerometer	Dynamic	Custom (a.10 healthy subjects; 8 activities b.10 elderly subjects; 8 activities)	Threshold	YES
Chen et al. (2018)	Fall Detection	Tri-axial accelerometer embedded in a smartphone	Static	Custom (10 healthy subjects; 13 activities)	SVM	NO
Islam et al. (2017)	Fall Detection	Tri-axial accelerometer embedded in a smartphone	Dynamic	Custom (7 healthy subjects; 4 activities)	Threshold	NO
Medrano et al. (2017)	ADL and Fall Detection	Tri-axial accelerometer embedded in a smartphone	Static	tFall (Medrano et al., 2014)	Threshold	NO
Garcia-Ceja et al. (2018)	ADL and Fall Detection	Sound and accelerometer data embedded in a smartphone and a wrist-band	Static	Custom (3 healthy subjects; 7 activities) Berkeley MHAD (Ofli et al., 2013), UTD-MHAD (Chen et al., 2015) and Opportunity (Roggen et al., 2010)	Random Forest	YES
Torti et al. (2019)	Fall Detection	Tri-axial accelerometer	Dynamic	SisFall (Sucerquia et al., 2017)	LSTM	NO
Cola et al. (2017)	Fall Detection	Barometer	Dynamic	Custom (6 subjects; 9 activities)	Threshold	NO
Jung et al. (2015)	ADL and Fall Detection	Tri-axial accelerometer	Dynamic	Custom (N. A. subjects; 5 activities)	Threshold	NO
Hemmatpour et al. (2017)	ADL	Tri-axial accelerometer embedded in a smart-watch	Static	Custom (22 subjects; 2 activities)	Multilayer Perceptron	NO
Sheikh and Jilani (2023)	Fall Detection	Tri-axial accelerometer and gyroscope	Static	SisFall (Sucerquia et al., 2017)	SVM	NO
Goh et al. (2021)	ADL	Tri-axial accelerometer and gyroscope	Dynamic	MotionSense (Malekzadeh et al., 2018), UCI-HAR (Garcia-Gonzalez et al., 2020) and USC-HAD (Zhang and Sawchuk, 2012)	1D-CNN and LSTM	YES
Luwe et al. (2022)	ADL	Tri-axial accelerometer and gyroscope	Dynamic	MotionSense (Malekzadeh et al., 2018), UCI-HAR (Garcia-Gonzalez et al., 2020) and custom	1D-CNN and LSTM	YES
Kumar and Suresh (2023)	ADL	IMU: tri-axial accelerometer, tri-axial gyroscope and tri-axial magnetometer	Dynamic	WISDM (Kwapisz et al., 2011), PAMAP2 (Reiss and Stricker, 2012) and KU-HAR (Sikder and Nahid, 2021)	CNN and RNN	YES
de la Cal et al. (2023)	ADL and Fall Detection	Tri-axial accelerometer embedded in a wrist-band	Static	UMAFall (Casilari et al., 2017), UCIFall (Özdemir and Barshan, 2014) and FallAllD (Saleh et al., 2021)	KNN and K-Means	NO
Warunsin and Phairoh (2022)	ADL and Fall Detection	Tri-axial accelerometer	Static	MobiAct (Vavoulas et al., 2016)	Multilayer Perceptron	NO
Hybrid solutions						
Clapés et al. (2018)	ADL	Two RGB-depth cameras, accelerometer, gyroscope, and magnetometer	Static	Custom (14 elderly subjects; 6 activities)	SVM	YES
Geman and Costin (2014)	ADL	RGB-depth camera and tri-axial accelerometer	Static	Custom (88 subjects; 3 activities)	Multilayer Perceptron	YES
Kwolek and Kepski (2014)	Fall Detection	RGB-depth camera and tri-axial accelerometer	Dynamic	URFD (Kwolek and Kepski, 2014)	Threshold	YES
Non-wearable Solutions						
Fan et al. (2020)	ADL	Radio-frequency sensors embedded in the floor and RGB camera	Dynamic	Custom (N. A. subjects; 157 activities)	Attention- based LSTM	YES
Chen et al. (2021)	ADL	WiFi sensor network	Dynamic	Custom (1 subject; 7 activities)	SVM and GRU	YES
Zerrouki et al. (2018)	ADL	RGB cameras	Dynamic	URFD (Kwolek and Kepski, 2014) and UMAFall (Casilari et al., 2017)	AdaBoost	YES
Karayaneva et al. (2023)	ADL	Low-resolution infrared camera	Static and Dynamic	Custom (6 subjects; 15 activities)	SVM, random forest, k-NN, logistic regression, and convolutional LSTM	YES
Mashiyama et al. (2015)	ADL and Fall Detection	Low resolution infrared array sensor	Static	Custom (N. A. subjects; 5 activities)	Threshold-based SVM	NO
Spasova et al. (2016)	Fall Detection	Low resolution infrared array sensor	Static	Custom (5 subjects; 2 activities)	SVM	NO
Roland et al. (2018)	Drinking	Tri-axial accelerometer embedded in a cup	Static	Custom (N. A. subjects; 1 activities)	Multilayer Perceptron	YES
Chaccour et al. (2015)	ADL and Fall Detection	Piezoresistive pressure sensors	Static	Custom (3 subjects; 6 activities)	Threshold	NO
Yue et al. (2020)	Sleeping postures	FMCW radio equipped with an antenna array	Static	Custom (26 subjects; 4 activities)	Multilayer Perceptron	YES
Li and Sun (2021)	ADL	RGB-depth cameras	Dynamic	Florence3D-Action (Seidenari et al., 2013), Toyota Smarthome (Das et al., 2019) and NTU RGB + D (Shahroudy et al., 2016)	CNN	YES
Srimath et al. (2021)	ADL	RGB-depth cameras	Dynamic	UTD-MHAD (Chen et al., 2015), KTH (Schuldt et al., 2004) and UCF-Sports (Rodriguez et al., 2008)	1D-CNN	YES
Tu et al. (2018)	ADL	RGB-depth cameras	Dynamic	SmartHome (Liu et al., 2017) and NTU RGB + D (Shahroudy et al., 2016)	3D-CNN	YES
Ma et al. (2018)	Hand gestures	RGB-depth camera	Dynamic	Custom (14 subjects; 28 activities)	LSTM	YES
Cippitelli et al. (2016)	ADL	RGB-depth camera	Static	KARD (Gaglio et al., 2015), CAD-60 (Sung et al., 2012), UTKinect (Xia et al., 2012), Florence3D-Action (Seidenari et al., 2013) and MSR Action3D (Li et al., 2010)	SVM	YES
Liu et al. (2020)	Hand gestures	RGB-depth camera	Static	Custom (30 subjects; 15 activities) and MSRA hand gesture (Sun et al., 2015)	3D-CNN	YES
Devanne et al. (2019)	ADL	RGB-depth camera	Dynamic	Watch-n-Patch (Wu et al., 2015)	LSTM	YES
Guerra et al. (2023)	ADL	RGB-depth camera	Dynamic	Custom (12 subjects; 4 activities)	GRU	YES
Guerra et al. (2022)	ADL	RGB-depth camera	Dynamic	Custom (12 subjects; 5 activities)	LSTM	YES
Guerra et al. (2020)	ADL	RGB-depth camera	Static	Custom (12 subjects; 3 activities)	Multilayer Perceptron	YES
Madhu et al. (2021)	ADL	RGB-depth camera	Static	MSR Action3D (Li et al., 2010)	Threshold-based SVM	YES
Su et al. (2017)	ADL	RGB-depth camera	Static	MSR Action3D (Li et al., 2010)	SVM	YES
Gonçalves et al. (2023)	ADL	RGB-depth camera	Static	Custom (15 subjects; 3 activities)	CNN	NO
Siriwardhana et al. (2019)	ADL	RGB-depth camera	Dynamic	Custom (17 subjects; 24 activities)	CNN-LSTM	YES
Poulose et al. (2022)	ADL	RGB camera	Static	Custom (10 subjects; 9 activities)	CNN	NO

## 5. Conclusion

In conclusion, it is significant to look toward the future of AAL systems, giving importance to HAR indoors and outdoors. The outdoor environment offers numerous activities that can contribute to preventing functional decline in frail individuals. However, monitoring outdoor activities presents different challenges, as non-wearable sensors may not be suitable and safety risks are increased. Therefore, wearables become crucial in this context. While user acceptance can sometimes be a challenging requirement to meet, the ease of use and unobtrusiveness of wearables greatly overcome this drawback. Wearable sensors enable continuous monitoring of indoor and outdoor activities, allowing for a more comprehensive assessment of an individual’s daily life. By incorporating wearable technology into AAL systems, it becomes possible to gather valuable data and insights to support preventive measures and promote healthy lifestyles among frail individuals.

As research and technological advancements continue, it is important to explore and optimize the use of wearable sensors in AAL systems, considering the specific constraints and requirements posed by outdoor monitoring. Future research trends in wearables design could be an enhanced miniaturization of the sensors used nowadays leading to better unobtrusiveness and the possibility of integrating these sensors inside even smaller devices or directly into clothes. An example is the fast development of Micro Electro-Mechanical Systems (MEMS) which enabled the optimization of several sensors-based applications. Moreover, power efficiency will represent a crucial issue, since it impacts both on communication and battery life. Improvements on battery technology should also be coupled with energy harvesting methods to partially recharge the device. On the other hand, the technological evolution of processors and microcontrollers could enable the adoption of state of the art classification methods, overcoming the actual limitations on the models size related to the available amount of memory and of computational power. Finally, communication technologies such as 6G could represent the ideal technology to transmit data between acquisition points and data collection centers. By doing so, the effectiveness of these systems can be enhanced in supporting independent living, improving safety, and preventing functional decline in the target population.

## Author contributions

BG: Conceptualization, Investigation, Writing - original draft. ET: Conceptualization, Investigation, Writing – original draft. EM: Conceptualization, Investigation, Writing – original draft. MS: Writing – review & editing. SR: Writing – review & editing. FL: Writing – review & editing. GD: Writing – review & editing.

## Footnotes

^1^ https://www.beghelli.it/salvalavita

^2^ http://www.lifealert.com

^3^ https://www.tensorflow.org/lite/microcontrollers

^4^ https://www.st.com/content/st_com/en.html

^5^ https://www.tensorflow.org

^6^ https://keras.io

^7^ https://pytorch.org

^8^ https://www.edgeimpulse.com

^9^ https://www.st.com/en/development-tools/nanoedgeaistudio.html
